# ASIC3, an acid-sensing ion channel, is expressed in metaboreceptive sensory neurons

**DOI:** 10.1186/1744-8069-1-35

**Published:** 2005-11-23

**Authors:** Derek C Molliver, David C Immke, Leonardo Fierro, Michel Paré, Frank L Rice, Edwin W McCleskey

**Affiliations:** 1 Vollum Institute, Oregon Health and Science University, Portland, Oregon 97239, USA; 2 Center for Neuropharmacology and Neuroscience, Albany Medical College, Albany, NY 12208, USA; 3 UPMC, Dept. Medicine, Univ of Pittsburgh, Pittsburgh PA 15261, USA; 4 Amgen, Inc., Dept of Neuroscience, One Amgen Way. Thousand Oaks CA. 91320, USA; 5 Dept de Ciencias Fisiologias, Universidad del Valle, Cali, Colombia; 6 AstraZeneca R&D Montreal, Montreal QC H4S 1Z9, Canada

## Abstract

**Background:**

ASIC3, the most sensitive of the acid-sensing ion channels, depolarizes certain rat sensory neurons when lactic acid appears in the extracellular medium. Two functions have been proposed for it: 1) ASIC3 might trigger ischemic pain in heart and muscle; 2) it might contribute to some forms of touch mechanosensation. Here, we used immunocytochemistry, retrograde labelling, and electrophysiology to ask whether the distribution of ASIC3 in rat sensory neurons is consistent with either of these hypotheses.

**Results:**

Less than half (40%) of dorsal root ganglion sensory neurons react with anti-ASIC3, and the population is heterogeneous. They vary widely in cell diameter and express different growth factor receptors: 68% express TrkA, the receptor for nerve growth factor, and 25% express TrkC, the NT3 growth factor receptor. Consistent with a role in muscle nociception, small (<25 μm) sensory neurons that innervate muscle are more likely to express ASIC3 than those that innervate skin (51% of small muscle afferents vs. 28% of small skin afferents). Over 80% of ASIC3+ muscle afferents co-express CGRP (a vasodilatory peptide). Remarkably few (9%) ASIC3+ cells express P2X3 receptors (an ATP-gated ion channel), whereas 31% express TRPV1 (the noxious heat and capsaicin-activated ion channel also known as VR1). ASIC3+/CGRP+ sensory nerve endings were observed on muscle arterioles, the blood vessels that control vascular resistance; like the cell bodies, the endings are P2X3- and can be TRPV1+. The TrkC+/ASIC3+ cell bodies are uniformly large, possibly consistent with non-nociceptive mechanosensation. They are not proprioceptors because they fail two other tests: ASIC3+ cells do not express parvalbumin and they are absent from the mesencephalic trigeminal nucleus.

**Conclusion:**

Our data indicates that: 1) ASIC3 is expressed in a restricted population of nociceptors and probably in some non-nociceptors; 2) co-expression of ASIC3 and CGRP, and the absence of P2X3, are distinguishing properties of a class of sensory neurons, some of which innervate blood vessels. We suggest that these latter afferents may be muscle metaboreceptors, neurons that sense the metabolic state of muscle and can trigger pain when there is insufficient oxygen.

## Background

ASICs are ion channels that open when the extracellular pH drops [1,2]. They belong to the epithelial sodium channel/degenerin superfamily of ion channels [3,4]. Members of this family are selective for sodium, are inhibited by the diuretic drug amiloride, and have two membrane-spanning regions with intracellular N- and C-termini and a large extracellular loop; multiple subunits must assemble to form a functional ion channel. There are three known ASIC genes that can form ion channels and two splice variants. The mRNA for all these ASICs are found in sensory ganglia; ASIC3 is unique in that, in rodents, little or none of it is expressed outside of sensory neurons [5].

The present study asks whether the distribution of ASIC3 among subsets of sensory neurons is consistent with its hypothesized roles as a transducer of two distinctly different sensations: ischemic pain and mechanosensation. ASICs were first considered as possible mechanosensitive ion channels because of their structural similarity to degenerins, which subserve touch sensitivity in *Caenorhabditis elegans *[6]. In rodents, ASICs are expressed in specific skin mechanosensory endings [7-9] and ASIC knockout mice exhibit subtle behavioral changes in their touch sensitivity in some assays [9-11], but not in others [12,13]. ASIC3 may act as a sensor of the lactic acidosis that occurs during ischemic pain because: a) it generates exceedingly large currents in dorsal root ganglion (DRG) sensory neurons that innervate the heart [14,15], an organ whose only conscious sensation is ischemic pain (angina); b) it responds to the small change in extracellular pH (a drop from 7.4 to 7.0) that occurs in the extracellular space of metabolically stressed muscle [15]; c) it responds better to lactic acid than to other forms of acid [16]; d) multiple injections of acid into skeletal muscle induce a long-lived painful hypersensitivity, but this does not occur in mice that lack ASIC3 [17]. In contrast to a possible role in muscle pain, evidence from transgenic mice argues against ASIC3 directly triggering cutaneous pain [18], although ASICs appear to contribute to acid-induced pain in human skin [19,20].

Two prior studies explored ASIC3 immunoreactivity in sensory neurons. One found ASIC3 and ASIC2 to co-localize in dorsal root ganglion neurons and to be present more often in larger neurons than in smaller [21]. The other found ASIC3 in both large and small neurons of the trigeminal ganglion and to exhibit some co-expression with CGRP [22]. The present study extends these earlier ones by combining retrograde labeling, electrophysiology and immunocytochemistry to distinguish various populations of nociceptive and non-nociceptive sensory neurons. The results suggest that high ASIC3 expression is an essential feature of a specific subset of nociceptors that respond to the metabolic state of muscle, but this is not the only population of sensory neurons that express ASIC3.

## Results

### ASIC3 observed in both TrkA and TrkC-positive neurons

**Figure 1:**
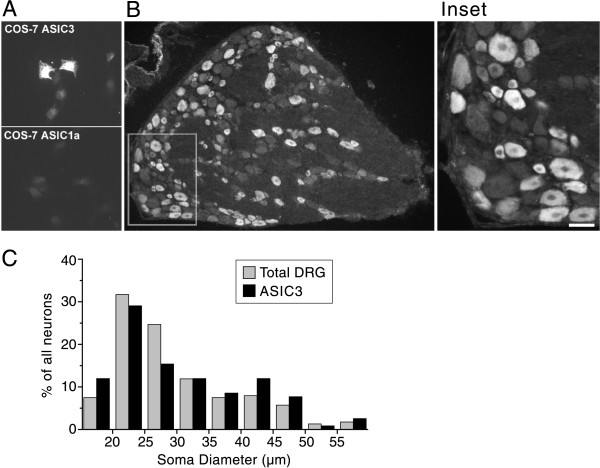
**Immunocytochemical localization of ASIC3 in rat sensory neurons**. A, ASIC3 antibody labeled COS-7 cells transfected with ASIC3 (top), but not those transfected with ASIC1a (bottom). It also failed to label cells transfected with ASIC1b and ASIC2a (not shown); ASIC2b and ASIC4 were not tested. B, ASIC3 immunoreactivity in a section of L5 DRG. Less than half of neurons are labeled and no non-neurons are labeled. Note the presence of both small and large labeled neurons (inset). Scale bar, 40 μm. C, Size-frequency distribution of ASIC3-positive neurons (black) and of all neurons (grey) from the L5 DRG. ASIC3+ cells are present in all size bins. 3345 neurons (12–15 sections from 3 rats) were counted, of which 1359 (40%) were ASIC3-positive.

**Figure 2:**
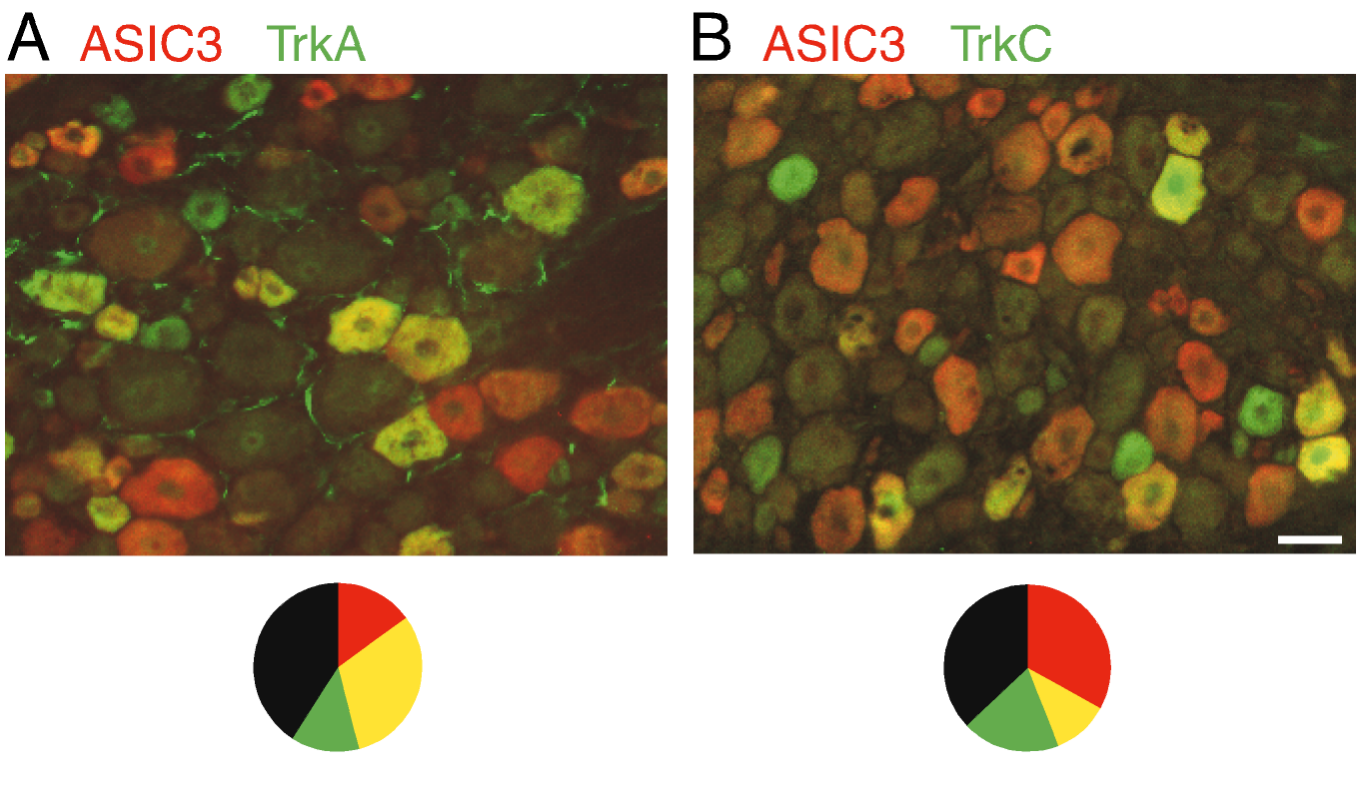
**ASIC3 co-expression with TrkA and TrkC**. Photomicrographs of DRG neurons labeled with antibodies to ASIC3 (red) and TrkA (A, green) or TrkC (B, green). Double-labeled cells appear yellow or orange. Circle charts show the fraction of double labeled cells (yellow), unlabeled (black), ASIC3-only (red), and TrkA or TrkC-only (green). 944 cells counted for A; 832 for B. Scale bar, 40 μm.

**Figure 3:**
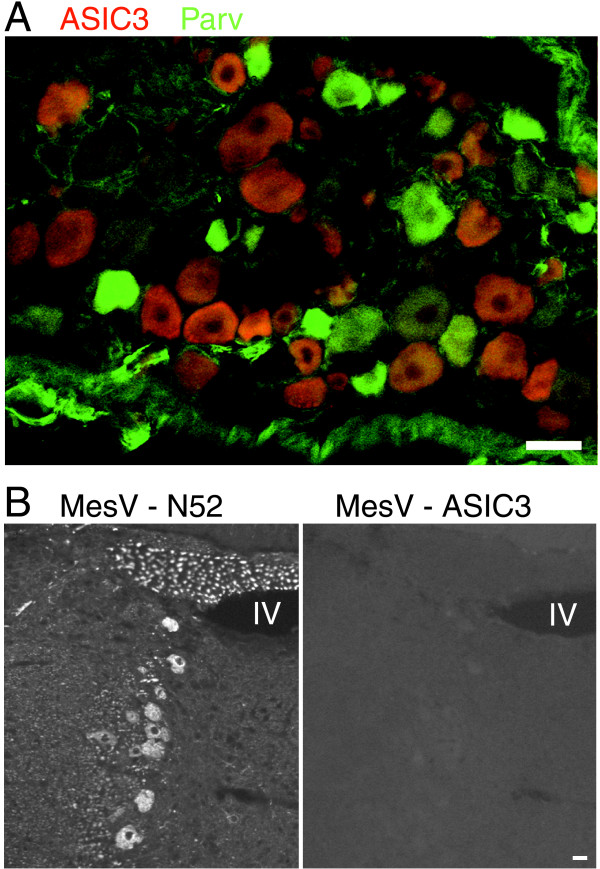
**ASIC3 is not expressed by muscle spindle afferents**. A, Section of DRG labeled for ASIC3 (red) and for the calcium-binding protein parvalbumin (green), which labels muscle spindle afferents. Only 2 of 1400 parvalbumin-positive cells had detectable ASIC3 staining. B, Section of brainstem containing a string of sensory neurons in the mesencephalic nucleus of the 5^th ^nerve (MesV) stained with either an antibody to a neurofilament protein (N52) to reveal all the sensory neurons (left, string of large cells arranged vertically; top punctuate labeling are MesV axons in cross section) or with the ASIC3 antibody (right). No MesV cells expressed ASIC3 (n = 3 rats). "IV" marks the fourth ventricle. Scale bars, 40 μm.

**Figure 4:**
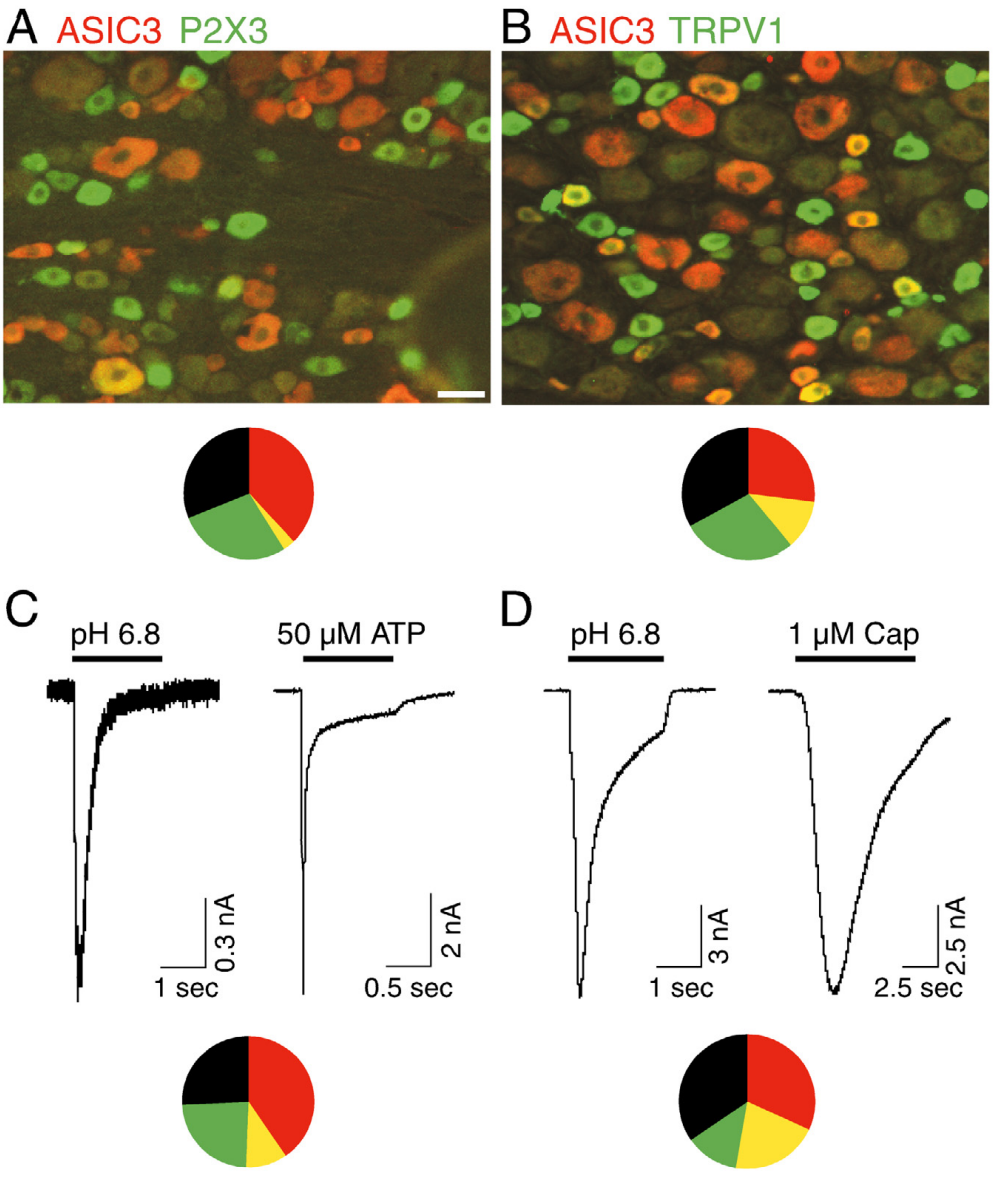
**ASIC3 expression compared to other nociceptive ion channels**. DRG sections double-labeled for ASIC3 (red) and either P2X3 (green, A) or TRPV1 (green, B); double-labeled cells are yellow or orange. Circle charts show the relative distribution of the three receptors among all DRG neurons. ASIC3 and P2X3 expression overlapped in only 4% of neurons, while some 35% of ASIC3-positive neurons also express TRPV1. The rare cells positive for both ASIC3 and P2X3 were large (eg. cell at lower left in A). 1406 cells counted for A; 995 for B. C, D, electrophysiological recordings reveal a qualitatively similar expression pattern for currents conforming to ASIC3, P2X3, or TRPV1. The cell that generated the currents in C counts as a co-expressor of ASIC3 and P2X3, fitting into the yellow bin in the circle plot. The cell in D is a co-expressor of ASIC3 and TRPV1. Cells were counted positive if they had at least 0.3 nA of current in response to pH 6.8 (ASIC3+), 50 μM ATP (P2X3+; transient current only), or 1 μM capsaicin (TRPV1). 182 cells recorded for C; 169 for D.

**Figure 5:**
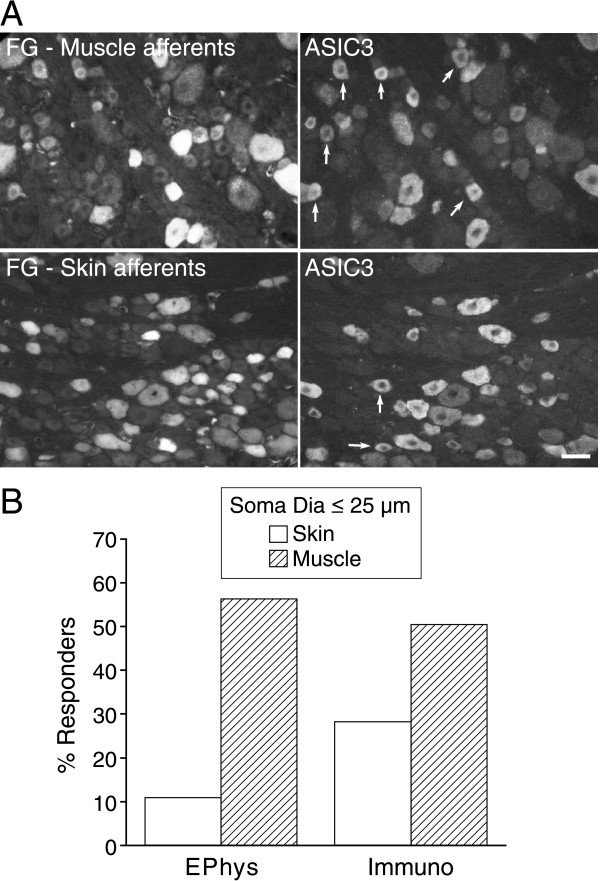
**Muscle nociceptors more likely express ASIC3 than do skin nociceptors**. *A*, Photomicrographs on the left and right are the same DRG section photographed for ASIC3 immunoreactivity (right) and fluorogold (left) that was retrogradely transported from injections into muscle (top) or skin (bottom). Arrows indicate cells that are 25 μm or smaller and contain both fluorogold and ASIC3 immunoreactivity. Scale bar, 40 μm. *B*, The percent of small (<25 μm) muscle or skin afferents that stain for ASIC3 (Immuno) or exhibit more than 0.3 nA of current evoked by a step to pH 6.8 from pH 7.4 (Ephys). 37 and 32 small neurons were recorded for skin and muscle, respectively. 117 and 113 small neurons were counted for skin and muscle immunocytochemistry.

**Figure 6:**
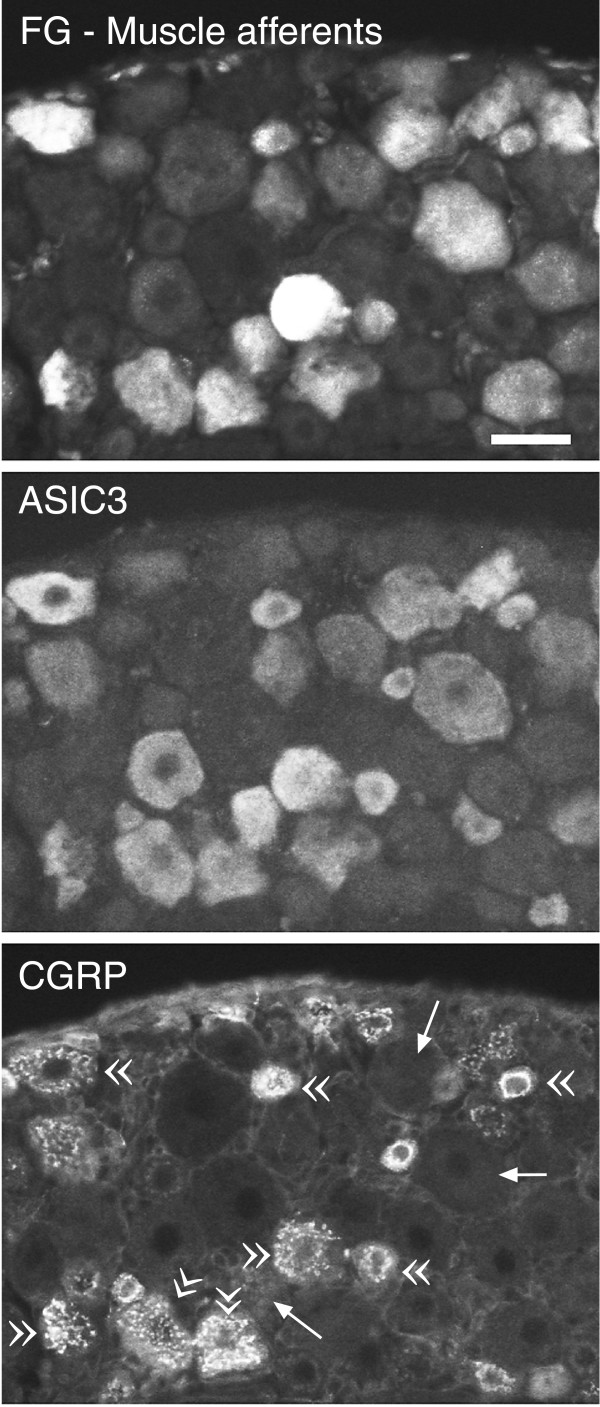
**Most muscle afferents that express ASIC3 also express CGRP**. Three photomicrographs of the same DRG section showing: fluorogold transported from a muscle injection (top); ASIC3 immunoreactivity (middle); CGRP immunoreactivity (bottom). Double arrowheads in the bottom image indicate cells positive for each label. Arrows indicate cells positive for fluorogold and for ASIC3, but not for CGRP. 83% (168/202) of ASIC3-positive muscle afferents were CGRP-positive (373 total muscle afferents counted). Scale bar, 40 μm.

**Figure 7:**
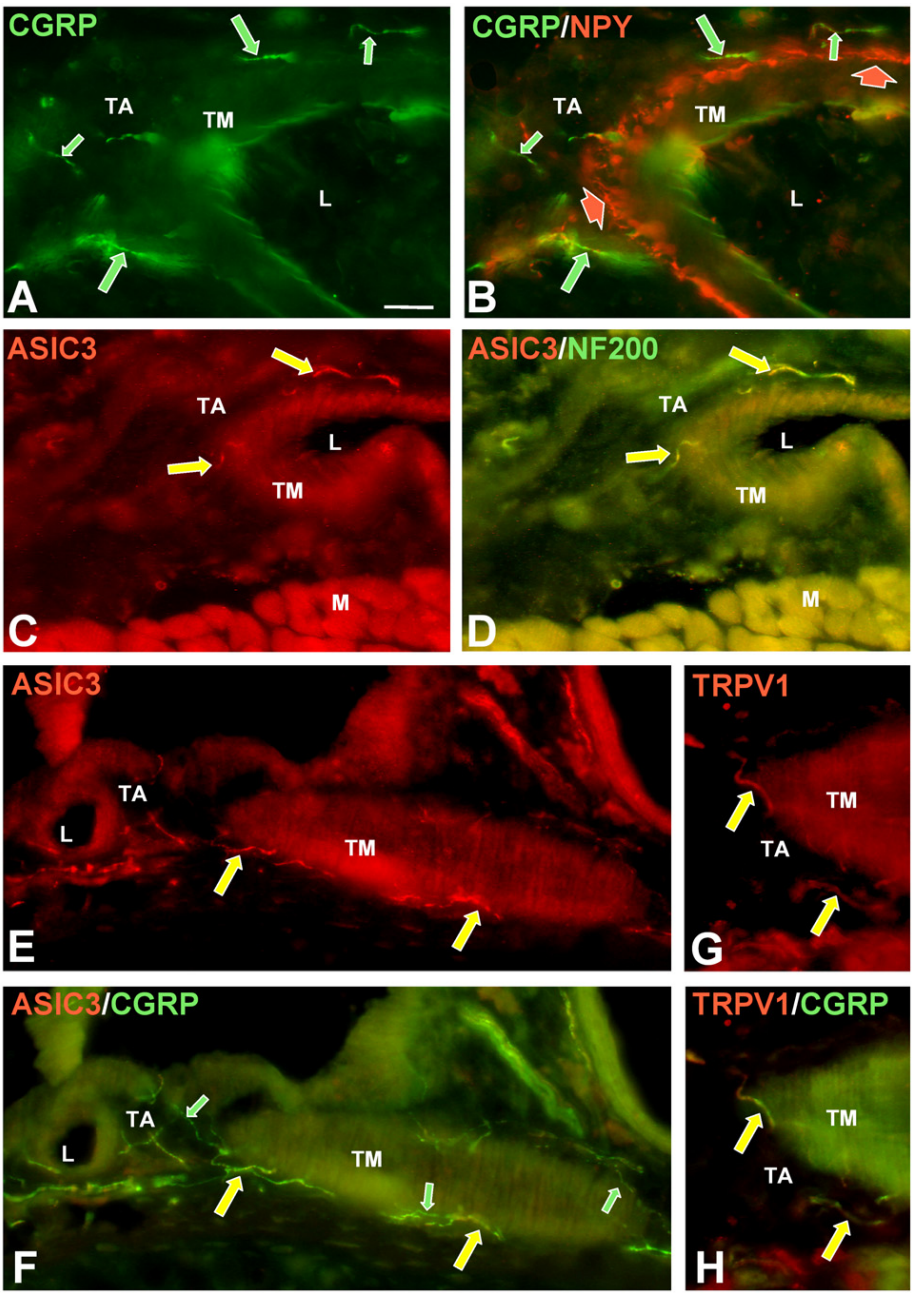
**Double-label immunofluorescence of arterial innervation among muscles in the lateral plantar compartment of the rat hind paw**. Red labeling is with secondary antibodies conjugated with Cy3, green with Cy2. The images in A and B are from a larger source artery and those in C-H are from smaller tributaries. L = arterial lumen, TA = tunica adventitia, TM = tunica media, M = skeletal muscle. Scale bar = 25 μm. A, B. Cross section of a larger artery with lumen (L) toward the right. Anti-CGRP (A) labeled sensory innervation that is located primarily in the tunica adventitia (green arrows). This CGRP-positive sensory innervation is supplied by axons that have thin calibers (small green arrows) and slightly thicker-calibers (large green arrows). Double labeling with anti-NPY (B) revealed sympathetic innervation concentrated at the border between the tunica media and tunica adventitia (red arrows). C, D. Cross section of an arteriole with lumen to the upper right, Muscle fibers (M) in cross section below. Double staining with anti-ASIC3 (C) and anti-NF200 (D) demonstrated that ASIC3 is expressed primarily on the slightly thicker caliber innervation that is NF200-positive (yellow arrows). E, F. Small arterioles viewed both in cross section (lumen labelled L) and on edge (oval structure labelled TM). Double labeling with anti-ASIC3 (E) and anti-CGRP (F) revealed that ASIC3 is coexpressed exclusively with CGRP, primarily on the thicker-caliber innervation (yellow arrows). The smaller-caliber CGRP innervation is usually ASIC3-negative (green arrows). G, H. Edge of an artery cross section; lumen is out of the picture to the right; tunica media (TM) and tunica adventitia (TA) are marked. Double labeling with anti-TRPV1 (E) and anti-CGRP (F) demonstrated that TRPV1 is also coexpressed on the larger caliber CGRP-positive innervation (yellow arrows).

The guinea pig ASIC3 antibody from Neuromics was used for all staining of dorsal root ganglia (DRG, Figs. 1, 2, 3, 4, 5, 6). We tested its specificity in COS-7 cells transfected with cDNA for the following ASICs: 1a, 1b, 2a, 3. It labelled ASIC3-transfected cells but not the others (Fig. 1A shows ASIC3 and ASIC1a; others are not shown but were negative like ASIC1a). No staining occurred if the antibody was preincubated with antigenic peptide or if the secondary antibody was applied without the primary (not shown). The antibody appears free of non-specific staining at the dilutions we used because it does not label tissue in the brainstem (Fig. 3B), which has no reported ASIC3 mRNA. Both guinea pig and rabbit anti-ASIC3 were used to label vascular innervation (Figure 7) and gave indistinguishable results.

Some 40% (1359/3345 cells in single label studies; 1932/4783 in double label) of DRG neurons stain for ASIC3 and there is no evident staining in non-neuronal cells or axon tracts (Fig. 1B). Both small (<25 μm diameter) and large (>40 μm) diameter sensory neurons express ASIC3. This contrasts with TRPV1, which is almost exclusively in smaller neurons [23]. The wide size distribution (Fig. 1C) suggests that anti-ASIC3 may label neurons that differ in conduction velocity and perhaps in sensory modality [24-26].

The TrkA receptor (the binding site for nerve growth factor) and the TrkC receptor (binding site for NT3 growth factor) label non-overlapping populations of sensory neurons [27,28]. The majority (68%, 284/417) of ASIC3+ neurons co-expressed TrkA (Fig. 2A). TrkA+ sensory neurons in adult rodents are considered to be NGF-sensitive nociceptors [29-31].

A minority (25%, 93/373) of ASIC3+ neurons co-expressed TrkC (Fig. 2B), a growth factor receptor used by many non-nociceptive mechanosensors [30]. Proprioceptors are among the sensory neurons that express TrkC [32]. Proprioceptors have two other testable properties: they express parvalbumin [33] and those from masticatory muscles have cell bodies in the brainstem at the mesencephalic trigeminal nucleus [34]. Only 2 of 1400 parvalbumin+ neurons co-expressed ASIC3 (Fig. 3A) and there was no staining in the mesencephalic nucleus (Fig. 3B). Evidently, ASIC3+/TrkC+ cells are not proprioceptors.

### ASIC3 co-expression with two nociceptive ion channels

To characterize the nociceptive population of neurons containing ASIC3, we tested for co-expression with two other ion channels thought to be pain-transducers: P2X3, an ATP-gated ion channel [35], and TRPV1, the capsaicin receptor that responds to noxious heat [36]. There was little overlap of ASIC3 and P2X3: although many neurons stained for one or the other, less than 5% (51/1406) stained strongly for both (Fig. 4A). We used electrophysiological recordings to test for expression of functional protein; only 10% of cells (18/182) had both ASIC3-like and P2X3-like currents that were larger than 0.3 nA (Fig. 4C). The cells that co-expressed ASIC3 and P2X3 were all large (examples: the one orange cell in Fig. 4A, lower left, and the cell (48 μm diameter) that gave the traces in Fig. 4C). Interestingly, virtually every large P2X3+ cell also stained positive for ASIC3, suggesting that this may be a distinct, albeit small population of sensory neurons.

There is more overlap between ASIC3 and TRPV1. 12% (122/995) of all cells stained for both, and 21% (35/169) had substantial current carried by both; 31% (122/388) of ASIC3+ cells were TRPV1+ (Fig. 4B,D). ASIC3/TRPV1 co-expressors were small (yellow cells, Fig. 4B), unlike the ASIC3/P2X3 co-expressors.

### More ASIC3 expression in small muscle afferents than small skin afferents

Although ischemic pain occurs when there is low blood flow to muscle, low flow to skin occurs routinely without any pain — for example, for thermoregulation or during the fight or flight response. Therefore, one might predict that, if ASIC3 is the sensor for ischemic pain, it should be in more muscle nociceptors than skin nociceptors. We tested this prediction by labelling muscle or skin afferents with retrogradely transported dyes, and then studying them with either electrical recordings or ASIC3 antibody staining; diI was used for electrophysiology and fluorogold for immunocytochemistry. In order to focus on nociception, we analyzed data for cells less than 25 μm diameter. However, as noted before, ASIC3 is also seen in many large neurons.

Figure 5 shows that small muscle afferents are more likely to express ASIC3 than are small skin afferents. A stimulus of pH 6.8, chosen to selectively stimulate ASIC3, elicited large current (>0.3 nA) in 56% (18/32) of small muscle afferents, but in only 11% (4/37) of small skin afferents (Fig. 5B). Immunocytochemistry presents a similar picture. The arrows in the right images of Fig. 5A point to small neurons (<25 μm) positive for both ASIC3 (right) and for fluorogold (left) transported from muscle (upper) or skin (lower); 50% (57/113) of small muscle afferents stained for ASIC3, compared to 28% (33/117) of small skin afferents (Fig. 5B).

### ASIC3 and CGRP co-express on sensory endings innervating blood vessels

Whether they have small diameters or large, most muscle afferents (83%, 168/173) that express ASIC3 also express the vasodilatory peptide, CGRP. The double arrowheads in the bottom image of Fig. 6 point to cells with evident nuclei that stained for all three labels: a) fluorogold that was previously injected into muscle (top panel); b) ASIC3 (middle); and c) CGRP (bottom). The arrows point to three ASIC3-positive muscle afferents that did not stain for CGRP. Overlap of CGRP and ASIC3 was also evident in skin afferents: 69% (168/244) of ASIC3 skin afferents also stained with the anti-CGRP.

Co-expression of ASIC3 with a vasodilatory peptide suggests that they might be expressed on sensory endings that innervate vasculature. Fig. 7 shows immunofluorescence images of arteries within rat lateral plantar muscles using the rabbit anti-ASIC3. L indicates the artery lumen, TA the tunica adventitia, and TM the tunica media. Nerve fibers that express CGRP are from sensory neurons [37]; they are relatively sparse and are present in the outer, adventitial layer (TA) of the artery wall (Fig. 7A). Fibers containing the neuropeptide NPY are from sympathetic neurons [37]; these fibers are denser than the CGRP fibers and lie at the border between the tunica adventitia and tunica media (TM) parts of the wall (Fig. 7B). Although all of the arterial innervation has a relatively small caliber, some CGRP-positive axons are noticeably thicker and are labeled by anti-200 kD neurofilament protein (NF200) and anti-myelin basic protein; this indicates that they are myelinated Aδ fibers [37]. The thinner CGRP-positive arterial innervation lacked NF200 and myelin basic protein immunoreactivity indicating that they are unmyelinated C fibers [37].

The ASIC3 antibody labels the thicker CGRP-expressing fibers in the tunica adventitia (large arrows, Fig. 7E,F) and it is co-expressed with NF200 (Fig. 7C,D). The thinner CGRP-expressing axons have little or no detectable ASIC3 immunoreactivity (small arrows, Fig. 7E,F). TRPV1 is also expressed on at least some of the thicker caliber CGRP-positive axons (Fig. 7G,H). In contrast to TRPV1 and ASIC3, antibodies to P2X3 do not label vascular afferents (not shown). In the adult, vascular CGRP-positive axons express TrkA but not TrkC [28].

## Discussion

We find ASIC3 in less than half of all DRG sensory neurons, but the population is diverse: ASIC3 can be in both small neurons and large, TrkA-expressors and TrkC. The neurons fall into at least two distinct subsets: 1) small to medium-size, TrkA- and CGRP-positive muscle afferents that lack P2X3 and can innervate small arteries; and 2) large neurons that lack parvalbumin and can express either TrkA or TrkC. We discuss the possibilities that neurons in the first subset are muscle metaboreceptors and the second includes non-proprioceptive mechanosensors.

### Metaboreception and the axon reflex

Because ASIC3 can open with quite small (0.5 unit) pH changes [15] and responds better to lactic acid than to other acids [16], it seems suitable for detecting when muscle is using anaerobic metabolism. Some consider metaboreception to be a subset of nociception [38]. In this view, low levels of metaboreceptor activity trigger compensatory responses in peripheral tissues and high levels trigger pain [39-41]. If ASIC3 is a molecular sensor for ischemia, it should be present in all cardiac DRG afferents (which, though diverse, all respond to cardiac ischemia, [42]), and it should be relatively enriched in sensory neurons that innervate muscle compared to skin because ischemic pain is characteristic of muscle but not skin. Our data here (on skin and muscle) and previously (on cardiac afferents, [14]) fulfill these expectations.

We found profound overlap of ASIC3 and the vasodilatory peptide CGRP. Over 80% of ASIC3-positive neurons that innervate muscle also express CGRP. Importantly, many sensory axons that innervate small arteries express ASIC3 together with CGRP and TRPV1. The axons that co-express the three markers appear to be lightly myelinated Aδ fibers because they express NF200, a marker of myelinated axons [43], whereas we saw no evidence that CGRP-positive small-caliber (C) arterial axons express ASIC3 or TRPV1. These ASIC3/TRPV1/CGRP-positive cells and axons uniformly fail to express P2X3, the primary ATP-gated ion channel in sensory neurons. Finally, the phenotype seems not to be unique to rats because ASIC3 labeling also occurs on CGRP-positive Aδ fibers that terminate on small arteries and arterioles in monkeys (Paré and Rice, unpublished). Extensive overlap of ASIC3 and CGRP has previously been reported in trigeminal ganglion neurons [22].

Release of CGRP in the periphery clearly contributes to the "axon reflex", in which activation of C fibers causes vasodilation and extravasation [44,45]. The tight co-expression of ASIC3, TRPV1 and CGRP on muscle arterial afferents raises the possibility that they function together as sensors and effector, increasing local blood flow in response to the presence of lactic acid and elevated muscle temperature. Co-expression of ASIC3 and TRPV1 should provide a cell with a broad range of acid sensitivity.

These observations add to a growing literature describing the phenotype of arterial afferents. They express TrkA immunoreactivity but not TrkC in adult mice and rats, though they express Trk C transiently during development [28]. The larger caliber arterial afferents express the histamine receptor 3, which is implicated in intense pressure mechanical nociception and allodynia [46].

We did not test for co-expression of ASIC3 with other ASIC subunits, but the existing literature addresses this well. ASIC3 and ASIC2, particularly the 2b splice variant, exhibit strong overlap [21]. ASIC1 appears expressed in a broader population of sensory neurons than is ASIC3 [21] and it is observed in many CGRP-expressing neurons [47]. The implication that ASIC3 and ASIC1 are often in the same cells is confirmed by both electrical and immunocytochemical observations in transgenic mice [48].

### Mechanosensation

Two labs have independently shown that ASICs are present in specific non-nociceptive mechanosensory nerve endings [7,9]. This includes ASIC3, with immunoreactivity observed in Aβ innervation that terminate as Merkel endings, Meissner corpuscle endings and hair follicle lanceolate endings in mice [10], as well as in rats and monkeys (Paré and Rice, unpublished). Data here indicates that 25% of ASIC3-staining cells also express TrkC, a growth factor receptor generally associated with non-nociceptive mechanosensors [30] and most clearly proven present in muscle proprioceptors [32]. However, the full range of sensory modalities among TrkC expressors is unknown. While it seems likely that the ASIC3+/TrkC+ cells include non-nociceptive mechanosensors, we cannot be certain.

We do know for certain that the ASIC3+/TrkC+ cells are not muscle spindle proprioceptors because they are absent from the mesencephalic trigeminal nucleus and they do not express parvalbumin. Other non-nociceptive mechanosensors show a diversity of expression patterns. Merkel cells and lanceolate endings do not express parvalbumin [49,50], lanceolate endings express CGRP [28,49-51], and Meissner's corpuscles express both CGRP and P2X3 [52]. Each of these expression patterns is observed in at least some of the ASIC3+ cells that we report. Thus, we have no data that conflicts with the expression pattern reported for skin mechansensory endings by Price and colleagues [10].

## Conclusion

Although less than half of all DRG sensory neurons express ASIC3, it is in multiple populations. One population has properties expected of metaboreceptors — cells that detect the metabolic state of muscle, mediate compensatory reflexes, and may initiate ischemic muscle pain. Over 80% of the ASIC3-containing muscle afferents co-express the vasodilatory peptide CGRP and some of these innervate small arteries; these cells seem poised to modulate blood flow in response to muscle stress. Another population of ASIC3-containing cells are quite large, can express TrkC, but do not express parvalbumin; this is a pattern observed with some non-nociceptive mechanoreceptors, but may not be unique to them.

## Methods

### Immunocytochemistry

Young adult Sprague-Dawley rats (150 g) were deeply anesthetized and perfused transcardially with 0.15 M phosphate-buffered saline, pH7.4 (PBS: 150 mM NaCl, 25 mM Na_2_HPO_4_, pH 7.4) followed by approximately 300 ml 3% paraformaldehyde with 15% picric acid in PBS or by 4% paraformaldehyde. The quality of ASIC3 staining was reduced with stronger fixation and in older rats, which exhibited unacceptable background fluorescence. Tissues were immediately dissected and placed in 30% sucrose in PBS overnight, then rapidly frozen and stored at -80°C until needed. Fourteen μm sections were cut on a cryostat and mounted on glass slides. Slides were placed in blocking solution containing 2% normal donkey serum, 0.2% Triton X-100 in PBS for 30 minutes, then incubated in primary antibody solution overnight at 4°C. Table 1 gives dilutions of the various antisera that were used. Next, slides were washed 3 times for 3 minutes in PBS and incubated in secondary antibodies diluted 1:200 in blocking solution for 30 minutes. Secondary antibodies were donkey anti-rabbit, donkey anti-mouse and donkey anti-guinea pig conjugated to CY3 or CY2 (Jackson Immunoresearch, West Grove PA). Slides were washed 3 times in PBS, mounted on coverslips in PBS and immediately photographed. For quantification, sections were taken from the fifth lumbar (L5) DRG. L5 was chosen because it is the ganglion used most commonly for immunocytochemistry. To minimize over-counting of large cells, only cells showing a clear nucleus were counted. All histograms and circle charts involved counts of over 1000 cells from at least three rats. Immunofluorescence assessments of arterial innervation were conducted on the lateral plantar artery and arterioles in the lateral plantar compartment of the foot. As described previously in detail [52], double labeling was conducted with various combinations of primary antibodies raised in different species.

**Table 1:** Antisera.

**Antisera**	**Species**	**Dilution**	**Source**
ASIC3	guinea pig rabbit	1:300 1:1000	Neuromics Robert Elde, University of Minnesota, Minneapolis
P2X3	rabbit	1:2000	Neuromics
TRPV1	rabbit guinea pig	1:1000 1:1000	Neuromics Neuromics
TrkA	rabbit	1:300	Chemicon, Temecula, CA
TrkC	Goat	1:300	Louis Reichardt, Universityof California, SF
Parvalbumin	mouse	1:1000	Sigma, St. Louis, MO
N52	mouse	1:10000	Sigma
CGRP	mouse rabbit guinea pig	1:2000 1:1000 1:500	RBI, Natick, MA Chemicon Peninsula
NPY	Sheep	1:1000	Chemicon
NF200	rabbit	1:1000	Chemicon

The guinea pig anti-ASIC3 was used to label all DRG (Figs. 1-6). Both guinea pig and rabbit anti-ASIC3 were used to label vascular innervation; when used together, each blocked labelling by the other. Fig. 7 shows results with the rabbit anti-ASIC3.

The Neuromics guinea pig ASIC3 antibody was used for all DRG cell body experiments (Figs. 1, 2, 3, 4, 5, 6). Both the guinea pig antibody and the rabbit ASIC3 antibody (generous gift of Robert Elde lab) were used to label vascular innervation (Figure 7 shows results with the rabbit antibody). The presence of either antibody blocked labelling by the other (not shown). The antibodies were raised against extracellular residues (237–257 for rabbit; 285–304 for guinea pig).

### Retrograde Labeling

DRG neurons innervating muscle and skin were labeled by injecting fluorescent dyes into either quadriceps muscle [53] or intradermally in the upper back between the shoulder blades. The intradermal injection raises a small blister that disappears within an hour; we did this in back skin because rats scratch and bite blisters put elsewhere, which would confound the assumption that dye is confined to the injection site. DiI (Molecular Probes, Eugene, OR. 5% in DMSO) was used for electrophysiology [54] and fluorogold (Fluorochrome Inc, Englewood CO. 1% in water) for immunocytochemistry because it withstands membrane permeabilization with detergent [55]. Dye was injected one to two weeks prior to sacrificing the rat.

### Electrophysiology

Whole cell patch clamp of dissociated sensory neurons measured currents carried by ASIC3, P2X3, or vanilloid receptors at -70 mV holding potential, room temperature. Solutions perfused the cells through 10 μl capillary pipettes and were changed within 20 msec with a computer-driven solenoid valve system. We used concentrations of ATP (50 μM) and capsaicin (1 μM) sufficient to evoke maximal responses in P2X3 and vanilloid receptors, but used a submaximal pH (6.8) stimulus. Because ASIC3 is more sensitive than other rat ASICs [15], pH 6.8 triggers ASIC3 channels without substantial activation of other subtypes. P2X3 currents were distinguished from other P2X receptors through their rapid desensitization. ATP-Na_2 _was added to the external solution from a 50 mM stock prepared in water at pH 7.4 (stored at -20°C). Capsaicin was prepared daily in ethanol.

Cervical or lumbar DRG were dissected and dissociated as previously described [14], plated on glass coverslips coated with poly-d-lysine and laminin, and maintained for approximately 2 hr at 37°C at 5% CO_2_/95% air in F12 media (Gibco BRL) plus 50 ng/ml nerve growth factor (NGF, Biomedical Tech. Inc.). Media was then changed to L15 (Gibco BRL) plus 50 ng/ml NGF at 23°C in humidified air. Electrophysiological recordings were made 24–48 hours after dissociation. Patch pipettes were pulled from borosilicate glass (Garner Glass, Claremont, CA) to 2.5–4 MΩ resistance. The standard internal solution contained (in mM): 135 methane sulphonic acid, 150 KOH, 10 KCl, 8 NaCl, 1 MgCl_2_, 10 MOPS, 0.3 Na_3_-GTP, 2 ATP-Mg, and 0.5 EGTA, adjusted to pH 7.0 with KOH. The control external solution contained (in mM): 140 NaCl, 5 KCl, 2 CaCl_2_, 1 MgCl_2 _10 HEPES, 10 MES, and 5 glucose, adjusted to pH 7.4 with n-methyl-d-glucamine; this was adjusted to pH 6.8 with HCl for the ASIC3-evoking solution. Data acquisition and analysis used PClamp 6.02 (Axon Instruments, Inc.) and Origin 6 (Microcal Software Inc). Chemicals were from Sigma Co. unless otherwise stated.

## Abbreviations

ASIC, acid sensing ion channel; ASIC3ir, ASIC3 immunoreactivity; CGRP, neuropeptide in sensory neurons that dilates blood vessels; DiI, a red lipophilic dye used for retrograde labeling of neurons; DRG, dorsal root ganglion; N200, neurofilament protein present mostly in myelinated axons; N52, neurofilament protein present in all sensory axons; NGF, nerve growth factor; NPY, neuropeptide Y; NT3, neurotrophin-3; ret, receptor for glial derived neurotrophic factor (GDNF); P2X3, ion channel opened by ATP; Trk A, nerve growth factor receptor; Trk C, neurotrophin-3 receptor; TRPV1 and VR1, ion channel opened by capsaicin, heat, or low pH; TA, tunica adventia; TM, tunica media

## Competing interests

The author(s) declare that they have no competing interests.

## Authors' contributions

DCM and DCI performed the immunocytochemistry on dorsal root ganglia. LF performed the electrophysiology. MP and FLR performed immunocytochemistry on vasculature. EWM oversaw the research and prepared the manuscript with help from all others.
